# Supplementary material for: Prenatal SARS-CoV-2 Infection Alters Human Milk-Derived Extracellular Vesicles
Source: Cells. 2025 Feb 15;14(4):284. doi: 10.3390/cells14040284 (PMC11853888; doi:10.3390/cells14040284)
Supplement: Supplementary file 1 [file cells-14-00284-s001.zip › Suppl figures_R1.pdf]

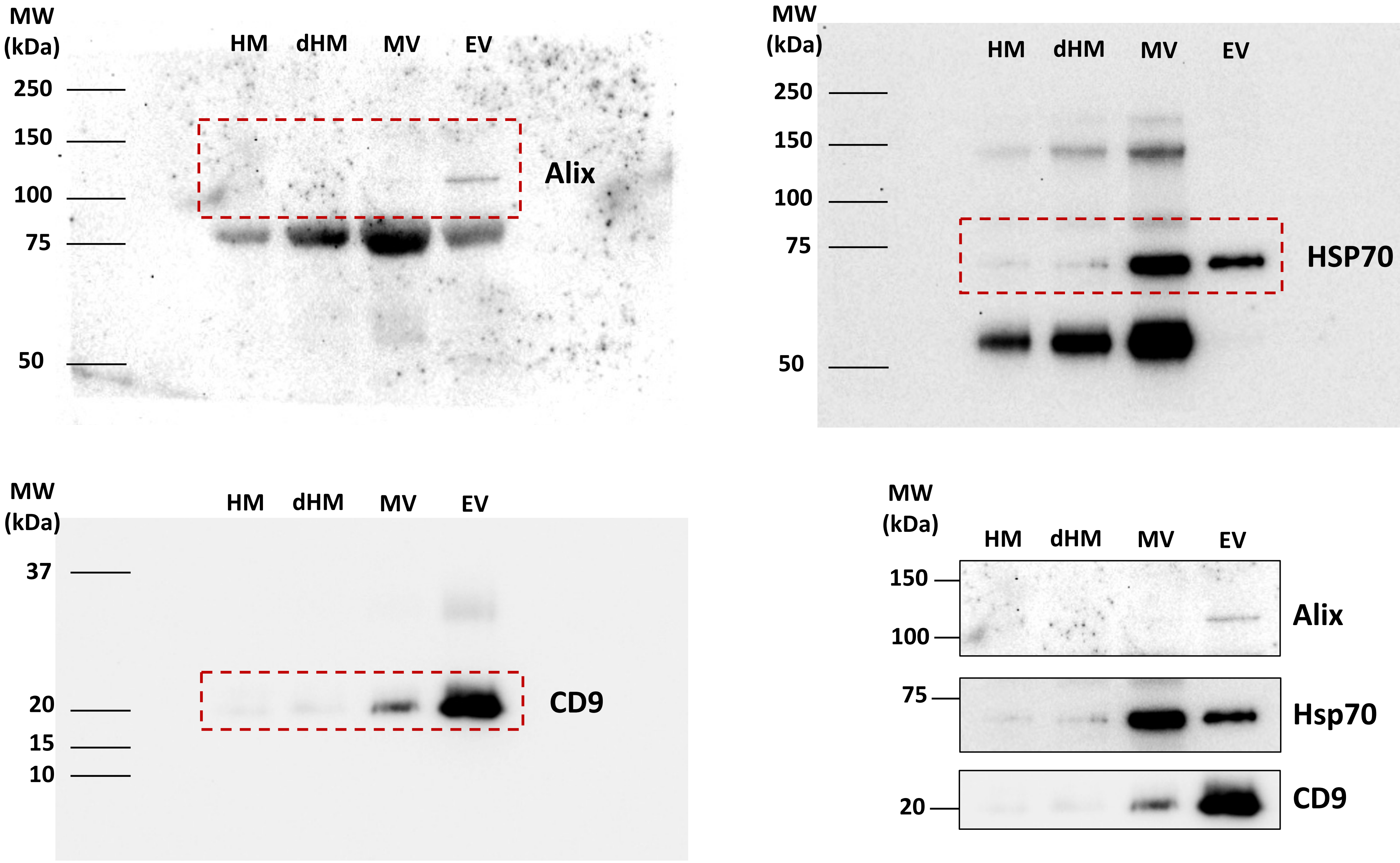

Figure S1. Full length blots of the cropped images presented in Figure 1d.

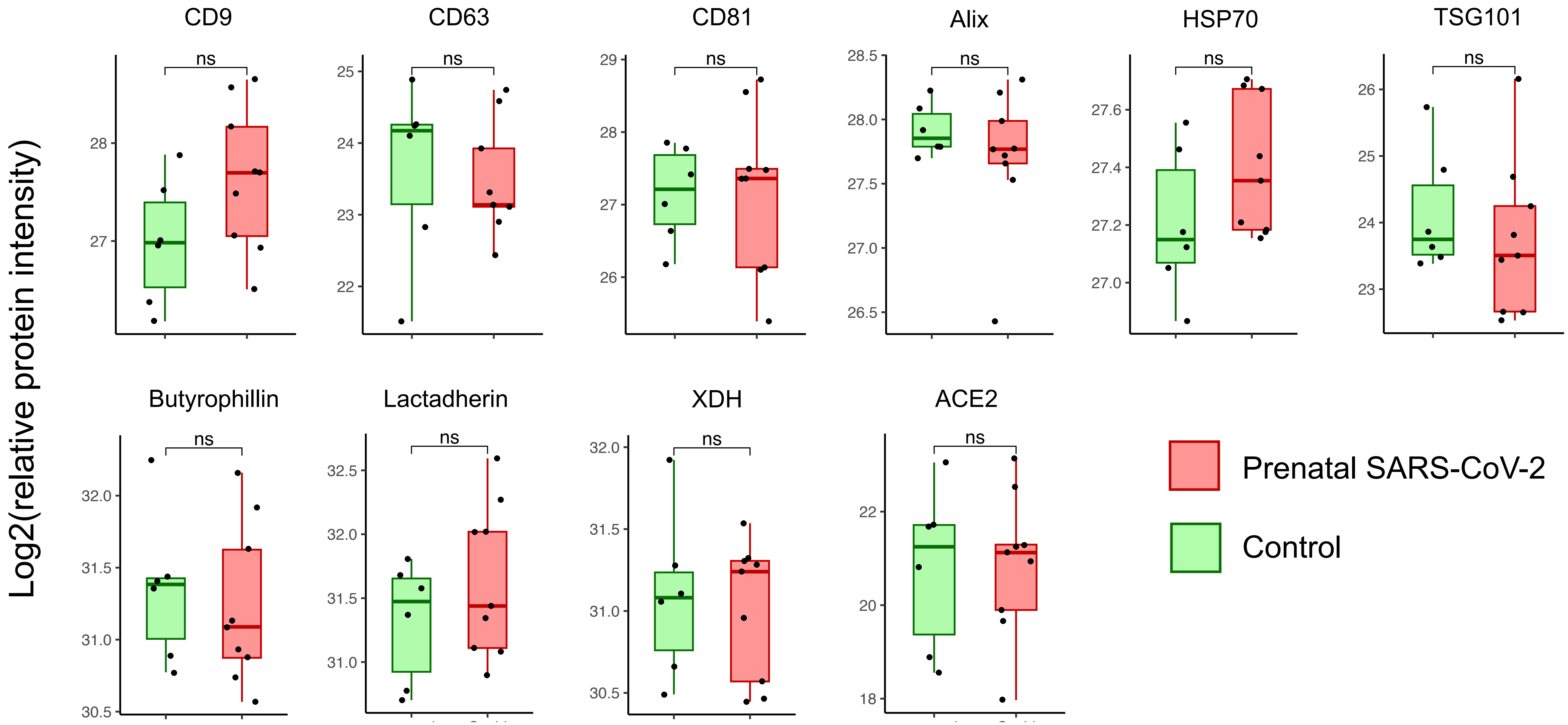

Figure S2. Relative protein intensities of common EV markers (tetraspanins CD9, CD63, CD81, Alix, HSP70, TSG101), HMEV markers (lactadherin, butyrophilin, XDH) and angiotensin converting enzyme 2 (ACE2) in HMEVs isolated from mothers with prenatal SARS-CoV-2 infection vs. controls.
